# Non-Response to Population Aging in Sub-Saharan Africa: A Survey of Gerontology Scholars

**DOI:** 10.1093/geroni/igab046.3029

**Published:** 2021-12-17

**Authors:** Margaret Adamek, Messay Kotecho, Samson Chane, Getachew Gebeyaw

**Affiliations:** 1 Indiana University, INDIANAPOLIS, Indiana, United States; 2 Addis Ababa University, Addis Ababa, Adis Abeba, Ethiopia; 3 Bahir Dar University, Bahir Dar, Oromiya, Ethiopia; 4 University of Gondar, Gondar, Oromiya, Ethiopia

## Abstract

Life expectancy is increasing globally, with the biggest gains expected in sub-Saharan Africa. In fact, most of the population growth globally in the next few decades will occur in sub-Saharan Africa. Using an online survey we investigated the perspectives of gerontology scholars on the challenges of aging in sub-Saharan Africa as well as the assets of elders. Respondents (n=72) from 17 countries, primarily in Africa, and representing 16 disciplines, identified the top issues facing African elders as: poverty, lack of trained professionals, food insecurity, disability/health issues, and long-term care. Older adults’ unique strengths were noted as indigenous knowledge systems, being holders of cultural heritage, and their contributions to development. Respondents’ biggest concerns about older adults in sub-Saharan Africa were the lack of government attention to aging issues (63%) and a lack of social services targeted to elders’ needs (57%). Government funding (77.8%) and international partnerships (38.9%) were noted as resources needed to support aging research in sub-Saharan Africa. The response or non-response of governments in sub-Saharan Africa will determine whether the growing number of older adults will increasingly experience unmet needs and whether their assets will be considered in development efforts. Establishing professional networks of gerontology scholars in the region will help to document the challenges faced by elders, to plan for the coming demographic shift, and to empower elders to thrive as valued community members.

